# Large‐scale Genome‐wide Investigation of Short Tandem Repeats Associated with Alzheimer’s Disease Risk

**DOI:** 10.1002/alz.091344

**Published:** 2025-01-03

**Authors:** Fahri Küçükali, Kristel Sleegers, EADB

**Affiliations:** ^1^ Department of Biomedical Sciences, University of Antwerp, Antwerp Belgium; ^2^ Complex Genetics of Alzheimer’s Disease Group, VIB Center for Molecular Neurology, VIB, Antwerp Belgium; ^3^ Complex Genetics of Alzheimer’s Disease group, VIB‐UAntwerp Center for Molecular Neurology, Antwerp Belgium

## Abstract

**Background:**

Classical genome‐wide association studies (GWAS) of Alzheimer’s disease (AD), which successfully identified over 75 risk loci to date, are limited to the content of the imputation panels that typically do not cover all types of genetic variation, e.g., tandem repeats encompassing >55% of human genome. Recent advances in the imputation panels with short tandem repeats (STRs; repeated motifs of 1‐6 bp in tandem, accounting for 10% of all repetitive elements) pave the way for large‐scale STR GWAS opportunities for complex diseases with substantial missing heritability such as AD. In this study, we investigated the contribution of STRs to AD risk.

**Method:**

We leveraged a publicly available STR imputation reference panel to impute over 440,000 multiallelic STRs in the European Alzheimer & Dementia Biobank (EADB) core cohort of 20,301 AD cases and 21,839 controls. After converting multiallelic STRs and applying filters of imputation quality score (R^2^≥0.3) and minor allele counts (≥20), imputed dosages from ∼2.35 million biallelic STRs were included in GWAS where the results were adjusted for the first 20 genetic principal components and genotyping center. We ran conditional analyses to determine independent risk loci driven by STRs. Furthermore, we constructed brain expression and splicing STR (eSTR & sSTR) catalogues for accurate mapping of non‐coding STRs to prioritized risk genes.

**Result:**

We identified 410 significant biallelic STR associations at *P*<1 × 10^‐5^, of which 194 were passing the stringent genome‐wide significant threshold, originating from 50 risk loci. ∼8% of the associations were rare (minor allele frequency <1%), while ∼10% of them were positioned outside of previously identified AD GWAS loci. One of these new association peaks was positioned near the promoter of a novel candidate gene, which was prioritized also by the eSTRs regulating the expression of this gene in the locus.

**Conclusion:**

Our first biallelic STR GWAS results demonstrate the power of our approach, and together with the complementary subsequent analyses (e.g., multiallelic length‐based STR GWAS method, conditional analyses, e/sSTR integration analyses, and additional replication and meta‐analyses), it has the potential to unravel and characterize AD‐associated STRs, towards a full characterization of all types of understudied genetic variation for AD in the future.

